# Clinical and experimental evidence suggest omicron variant of SARS-CoV-2 is inherently less pathogenic than delta variant independent of previous immunity

**DOI:** 10.1186/s40001-023-01373-3

**Published:** 2023-10-11

**Authors:** Ramachandran Thiruvengadam, Zaigham Abbas Rizvi, Sreevatsan Raghavan, Deepika Rathna Murugesan, Mudita Gosain, Jyotsna Dandotiya, Ayushi, Sweety Samal, Anil K. Pandey, Nitya Wadhwa, Shinjini Bhatnagar, Amit Awasthi, Pramod Kumar Garg

**Affiliations:** 1 Translational Health Science and Technology Institute, Faridabad, Delhi India; 2 ESIC Medical College and Hospital, Faridabad, India

**Keywords:** Omicron, SARS-CoV-2, COVID-19, hamster, hACE2 transgenic mice

## Abstract

**Objectives:**

To study clinical disease outcomes in both human and animal models to understand the pathogenicity of omicron compared to the delta variant.

**Methods:**

In this cross-sectional observational study, clinical outcomes of adults who tested positive at 2 testing centres in Delhi National Capital Region between January 2022 and March 2022 (omicron-infected; *N* = 2998) were compared to a similar geographical cohort (delta-infected; *N* = 3292). In addition, disease course and outcomes were studied in SARS-CoV-2-infected golden Syrian hamsters and K-18 humanized ACE2 transgenic mice.

**Results:**

Omicron variant infection was associated with a milder clinical course [83% (95% CI 61, 94) reduced risk of severity compared against delta] adjusting for vaccination, age, sex, prior infection and occupational risk. This correlated with lower disease index and vir comparing omicron with other variants in animal models.

**Conclusions:**

Infections caused by the omicron variant were milder compared to those caused by the delta variant independent of previous immunity.

## Introduction

The severe acute respiratory syndrome coronavirus 2 (SARS-CoV-2) B.1.1.529 variant, labelled as omicron, was first detected in South Africa in late November 2021 [1]. It has since rapidly spread globally, with over 1,841,834 confirmed cases as of 1st March 2022. India reported its first omicron case on 2nd December 2021. Although genetic and molecular characteristics of SARS-CoV-2 B.1.1.529 variant have been reported, its clinical characteristics and disease outcomes are not well-known. A few reports from South Africa and UK have shown a milder course of COVID-19 infection due to omicron in comparison with the delta variant particularly due to a lower incidence of viral pneumonia [1, 2]. It has been argued that acquired immunity following extensive vaccine coverage and prior natural SARS-CoV-2 infection could be the dominant reasons for the reduced population-level severity of omicron infection. Data from CDC, USA also suggests that omicron leads to somewhat milder disease compared to delta but the overall deaths are similar due to the higher infectivity rate of the omicron variant [3]. Thus, we aimed to study outcomes in both human and animal models to decipher the pathogenicity of the omicron variant. We compared the severity and clinical outcomes of omicron and delta infections in humans after adjusting for vaccination and natural infections. To discount the effect of acquired immunity and understand if the omicron variant is inherently less pathogenic or has less predilection for causing pneumonia, we further investigated the severity of delta and omicron infections in two animal models of SARS-CoV-2 infections.

## Materials and methods

### Clinical characteristics

#### Study design and participants

**Fig. 1:**
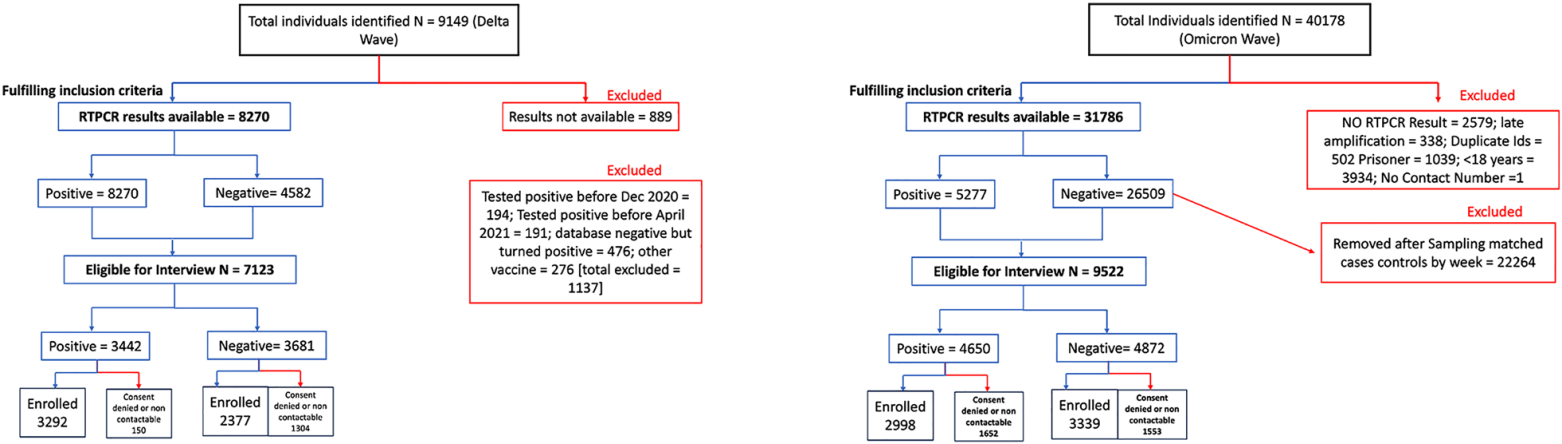
Study flow for the selection of the omicron cohort

In this cross-sectional observational study, we analysed the clinical characteristics of 2998 adult participants who tested positive (via RT-PCR) at the laboratories of Translational Health Science and Technology Institute, Faridabad, India and Employee State Insurance Corporation Medical College and Hospital between January 2022 and March 2022, coinciding with the surge dominated by the omicron variant in this geographical area (omicron cohort). These two centres contribute to over 90% of testing in the specified geographic region [4]. The omicron surge was confirmed by the population-level genome surveillance data maintained by INSACOG, Government of India, of which THSTI is a contributing member. The data showed that the proportion of omicron variant infections ranged from 92.03% (January 2022) to 100% (March 2022) in all the cases sampled during the study period (Fig. 1). These numbers were compared with a similar sample population taken during the surge driven primarily by the delta variant (March to May 2021) comprising of 3292 test-positive individuals (delta cohort) from the same testing centres as part of a previous vaccine effectiveness study, where the study flow was described in detail [4]. The cohorts were developed through the Department of Biotechnology (DBT) consortium for COVID-19 research which is an on-going initiative, conceived at the start of the pandemic in March 2020 with the aim of rapid collection, assessment and dissemination of scientific information in all aspects pertaining to the SARS-CoV-2 virus.

#### Data collection

Participants in both groups had their vaccination data (including vaccine name, number of doses, vaccination centre) and their respective clinical profile recorded during their respective disease course for features, such as presence of fever, sore throat, breathlessness, etc. This was recorded via telephonic interview by trained research staff with prior experience in such data collection. In addition, outcomes of severity like need for hospitalisation, oxygen therapy, mechanical ventilation and intensive care unit (ICU) admission with their respective associated days were also recorded. These were deemed as indicators of diseaase severity and progression.

Clinical Data Management and Quality control steps: The data were captured directly on electronic forms by the research team, and it was monitored real time by pre-applied quality checks for missing data and discrepant data.

1. The clinical data was collected telephonically hence real time call monitoring was established to ensure quality. Recall in 10% of cases was done by researchers for the participants if real time monitoring could not be completed.
2. Source data verification: the following source data were used for quality checks:
  - Name of Vaccine from available “vaccine certificate” shared by participant issued to them from the National COVID-19 vaccination (COWIN) portal or vaccination center
  - Date of vaccination dose from available “vaccine certificate” shared by participant issued to them from COWIN portal or vaccination center,
  - Date of RT PCR test from testing center.
  - Symptomatic/Asymptomatic status of participants who were from the DBT consortium

#### Severity assessment of omicron and delta SARS-CoV-2 infections in animal models

6–10-week-old golden Syrian hamsters were procured from National Institute of Nutrition (NIN, India) and quarantined for 1 week at small animal facility (SAF). K18-hACE2 transgenic mice (henceforth referred to as hACE2.Tg mice) were obtained from Jax Lab (US) and maintained at SAF. Hamsters (post quarantine) and 6–8-week-old hACE2.Tg mice were transferred to infectious disease research facility (IDRF) for Animal biosafety level-3 (ABSL-3) challenge study. The animals were housed under 12 h light and dark cycle and fed a standard diet ad libitum. All the experimental protocols, animal challenge and necropsy were approved by Translational Health Science and Technology Institute (THSTI) Institutional Animal Ethics Committee (IAEC), Institutional Biosafety (IBS) and Review Committee on Genetic Manipulation (RCGM).

#### Virus culture and titration

SARS-Related Coronavirus 2, Isolate USA-WA1/2020 virus (Wuhan strain), Isolate hCoV-19/USA/PHC658/2021 (delta variant) B.1.617.2 (NR-55611) and clinical isolate of SARS-CoV-2 variant Omicron (B.1.1.529) were grown and titrated in Vero E6 or Calu-3 cell line. The virus stocks were plaque purified at IDRF facility, THSTI inside ABSL3 following institutional biosafety guidelines.

#### SARS-CoV-2 infection

All animals were randomly allotted to different groups (*n* = 5) and assigned uninfected group (UI), Wuhan infection group (2019-nCoV), delta infection group (B.1.617.2) and omicron infection group (B1.1.529). All animals, except the uninfected control group, were challenged with respective virus strains inside ABSL3. Briefly, animals (6-8 weeks, mixed gender) were given intranasal infection with 10^5^ pfu virus/animal under mild anaesthesia condition as previously described [5–8]. Post-challenge the body mass of the animals was recorded daily for 14 days (for hamsters) or 6 days (for hACE2 mice). Hamsters were sacrificed at 4 day post-infection (dpi), while hACE2. Tg mice were sacrificed at 6 dpi. One set of hamsters was left till 14 dpi for body mass changes. All the animal challenge studies were approved by IAEC (protocol no. IAEC/THSTI/151), IBSC and RCGM.

#### Gross clinical parameters of SARS-CoV-2 infection

Lungs from the euthanized animals were excised and imaged for gross morphological changes. The right lower lobe of the lung was immediately fixed in a 10% neutral formalin solution and used for histological analysis. The remaining portion of the lung was homogenised in Trizol and used for RNA isolation and viral load estimation.

#### Histological analysis

Lungs: fixed lungs were processed and paraffin wax-embedded blocks were transverse sectioned and stained with hematoxylin and eosin (H & E) dye. The H & E stained lung sections were then quantitatively examined under the microscope for pneumonia, alveolar epithelial cell injury, inflammation, and lung injury on a scale of 0–5 by a trained pathologist who was blinded for the study. Scores for pneumonia, alveolar epithelial injury, lung injury, bronchitis and inflammation were given on a scale of 0–5, where 0 meant absence of feature, while 5 represented maximum injury. The disease index score was calculated as an average of the total score. Images of the H&E stained lung sections were acquired at 40X magnifications.

#### Viral load

Homogenised lung samples in Trizol were used for RNA isolation using the Trizol-chloroform method as per the manufacturer’s protocol. Isolated lung RNA was quantitated by NanoDrop and 1 µg of total RNA was then reverse-transcribed to cDNA using the iScript cDNA synthesis kit (Biorad; #1708891) (Roche). Diluted cDNAs (1:5) were used for qPCR using KAPA SYBR^®^ FAST qPCR Master Mix (5X) Universal Kit (KK4600) on Fast 7500 Dx real-time PCR system (Applied Biosystems) and the results were analysed with SDS2.1 software. CDC-approved SARS-CoV-2 N gene primers: 5′-GACCCCAAAATCAGCGAAAT-3′ (Forward), 5′-TCTGGTTACTGCCAGTTGAATCTG-3′ (Reverse) were used for vial load calculation. For absolute quantitation, the known copy number of the virus RNA was used as a standard to generate the calibration curve.

### Statistical analysis

For the human study, the clinical characteristics of the two respective virus surges in the population were compared using a logistic regression model factoring in gender, age, vaccination status, profession with high risk of exposure, and any history of previous natural SARS-CoV-2 infection, respectively. The analysis were conducted in R programming language. The animal model data was analysed and results were plotted using Graph pad prism 7.0 software. Body mass, gene expression, histology scores were compared and analysed using one-way ANOVA using Tukey’s test. A *P* value of less than 0.05 was considered as statistically significant.

### Results

The distribution of age (median age 32 vs 35; omicron vs delta, respectively), sex (omicron group—Males = 1089, Females = 1909; delta—Males = 1099, Females = 2193), and high risk participants were similar in both the omicron (*n* = 514) and delta (*n* = 496)-infected cohorts. As expected, the proportion of vaccinated individuals were higher in the omicron cohort: 2386 (80%) had received both doses of their vaccination and 471 (16%) had received their first dose of vaccination in comparison with the delta cohort: 234 (7.1%) had received both doses of their vaccination and 588 (18%) had received single dose (*P* < 0.001).

We observed a milder profile of COVID-19 during omicron-driven surge as compared with the delta-driven surge with only nine (0.3%) participants from omicron cohort requiring hospitalisation as compared to 167 (5.1%) in the delta cohort. Six patients required oxygen support with none requiring intensive care or ventilator support as compared to 99 (54% of the hospitalised subjects), 28 (16%), and 5 (2.8%), respectively, during the delta-driven surge. None in the omicron group and 12 (0.4%) patients in the delta cohort died. The detailed comparison is presented in Table 1.

**Table 1:** Clinical and demographic characteristics of participants infected during delta- and omicron-driven surges

Characteristic	Delta cohort, *N* = 3292	Omicron cohort, *N* = 2998	*P* value
Age	35 (28, 45)	32 (26, 42)	*P* < 0.01
Sex			*P* = 0.14
Female	1,099 (33%)	1089 (36%)	
Male	2,193 (67%)	1,909 (64%)	
Vaccination status			*P* < 0.001
Both Doses	234 (7.1%)	2,386 (80%)	
Single dose	588 (18%)	471 (16%)	
Unvaccinated	2,470 (75%)	141 (4%)	
Booster dose	0 (0%)	65 (2.7%)	
Risk of occupational exposure			*P* < 0.001
Defense Staff	123 (3.7%)	98 (3.3%)	
Essential Services	90 (2.7%)	59 (2.0%)	
Hospital Staff	229 (7.0%)	319 (11%)	
Municipal Worker	54 (1.6%)	38 (1.3%)	
None	2796 (85%)	2,484 (83%)	
Fever	2460 (74.7%)	1329 (44.3%)	*P* < 0.001
Cough	1210 (36.7%)	1456 (48.56%)	*P* < 0.001
Sore throat	1210 (36.7%)	1032 (34.4%)	*P* < 0.001
Breathlessness	506 (15.3%)	117 (3.9%)	*P* < 0.001
Headache	859 (26.1%)	412 (13.7%)	*P* < 0.001
Gastrointestinal symptom	463 (14.1%)	39 (1.3%)	*P* < 0.001
Comorbidity	408 (12.4%)	225 (7.5%)	*P* < 0.001
Hospitalisation	167 (5.1%)	9 (0.3%)	*P* < 0.001
Hospitalised subjects	*N* = 167	*N* = 9	*P* < 0.001
Days of Hospitalisation	9.0 (5.0, 10.0)	4.0 (3.0, 5.0)	*P* < 0.001
Oxygen Supplementation	99 (54%)	6 (66.7%)	*P* < 0.001
Duration of oxygen therapy	7.5 (5.0, 12.0)	6.0 (4.2, 9.2)	*P* < 0.001
ICU admission	28 (17%)	0 (0%)	*P* < 0.001
Duration of ICU stay	5 (2.5,7.0)	^a^Not applicable	
Ventilator support (N)	5 (3%)	0 (0%)	*P* < 0.001
Duration of ventilator support	3 (1.0, 9.5)	^a^Not applicable	
Death	12 (0.4%)	0 (0%)	*P* < 0.001

^a^No comparator available

The vaccine uptake at the population level was much higher during omicron-driven surge as compared to the delta-driven surge which might have significantly contributed to the reduction in the severity. Therefore, we adjusted for the difference in the vaccination rate, age, sex, prior SARS-CoV-2 infection and risk of occupational exposure in a multivariable regression and found that the omicron-driven infections were associated with 83% (95% CI 61, 94) reduced risk of severity. The same has been summarized in Table 2.

**Table 2:** Multivariate logistic regression model for the differences in clinical presentation between the delta and omicron cohort

Parameter	Adjusted odds ratio (OR)^a^	95% Confidence interval	*P* value
Clinical Severity^b^ of omicron variant (reference—clinical severity of delta variant)	0.13	0.04–0.30	< 0.001

^a^Odd ratio adjusted for gender, age, vaccination status, profession with high risk of exposure, and any history of previous natural SARS-CoV-2 infection, respectively

^b^Severity was defined as a composite outcome variable considering need for hospitalisation, oxygen therapy, mechanical ventilation and intensive care unit (ICU) admission along with the respective days associated with each parameter

**Fig. 2:**
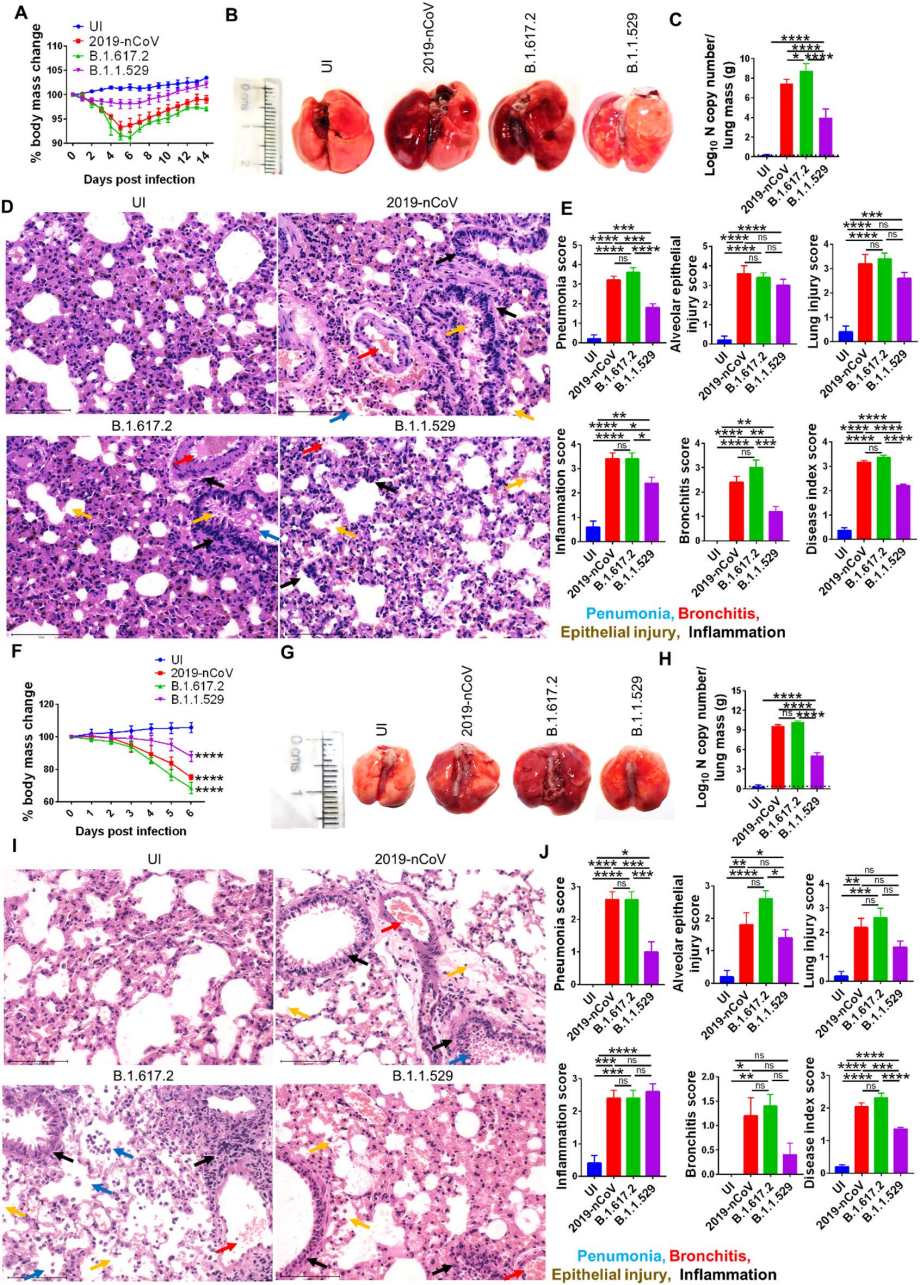
Pathological manifestations of Omicron (B.1.1.529) infection in Syrian hamster and hACE2.Tg mice. Pathological manifestations of intranasal Omicron (B.1.1.529) infection was evaluated and compared with ancestral Wuhan (nCoV-2019) and Delta (B.1.617.2) strain infection or uninfected (UI) in Syrian hamster and hACE2.Tg mice. The changes in body mass was plotted as percentage of the day 0 body mass till day 14 or 6 post infection, respectively, for (**A**) hamster and (**F**) hACE2.Tg mice. The lungs of the sacrificed animals were harvested and images and thereafter, viral load and histopathology of the lungs were studied. **B** and **G** Shows representative lungs from individual groups at 4 dpi and 6 dpi from hamster and hACE2.Tg mice, respectively. **C** and **H** Viral load of the lungs at 4dpi and 6 dpi from hamster and hACE2.Tg mice, respectively. **D**–**E** and **I**–**J** Histopathological assessment of the HE stained lungs were carried out by trained pathologist by blinded scoring on the scale of 0–5 (where 0 described no feature and 5 described highest pathological feature) of the lungs at 4 dpi and 6 dpi from hamster and hACE2.Tg mice, respectively. Each experiment was carried out with *n* = 5 animals and replicated 3 times independently. One-way ANOVA using non-parametric Kruskal–Wallis test for multiple comparison. ns = non-significant, ^*^P < 0.05, ^**^P < 0.01, ^***^P < 0.001, ^****^P < 0.0001

The disease course and outcomes of SARS-CoV-2 infection were also studied in golden Syrian hamsters and hACE2.Tg mice following intranasal challenge as previously described [5, 9]. The body mass of hamsters was recorded and plotted as a percentage change of day 0 body mass of the same animal. The percentage change in body mass data showed a gradual and comparable decrease in body mass of both ancestral Wuhan SARS-CoV-2 strain (2019-nCoV) and delta SARS-CoV-2 variant (B1.617.2) challenged hamsters. As compared to the uninfected (UI) control, hamsters challenged with Wuhan SARS-CoV-2 strain (2019-nCoV) and delta SARS-CoV-2 variant (B1.617.2) lost approximately 10% of body weight, while hamsters challenged with omicron SARS-CoV-2 variant (B.1.1.529) lost approximately 5% of body weight by 6-day post-infection (dpi). Post 6 dpi, animals from all the challenged groups continuously gained weight till 14 dpi suggesting a steady recovery from the disease (Fig. 2A). In line with changes in the body mass, the excised lungs of Wuhan or delta strain-infected hamsters showed prominent regions of inflammation and pneumonia as compared to the UI control lung at 4 dpi. However, the lungs of omicron-infected hamsters showed significantly lesser regions of inflammation and pneumonia (Fig. 2B). The lung viral load was estimated by N gene copy number and was found to be significantly higher in B.1.617.2 infection than in other SARS-CoV-2 strains infection groups at 4 dpi. Notably, B.1.1.529 infection resulted in two-folds lower (on log10 scale) lung viral load as compared to 2019-nCoV or B.1.617.2 infection in hamsters (Fig. 2C). Blinded assessment of lung histology showed a marked increase in pneumonia, alveolar epithelial injury, bronchitis, inflammation and overall lung injury scores in all the challenged groups as compared to UI control at 4 dpi. However, the overall disease index score of B.1.1529 infected animals were 25–30% lesser than the 2019-nCoV or B.1.617.2 infected animals (Fig. 2D, E). A challenge study was also performed in hACE2.Tg mice which is a lethal model for SARS-CoV-2 infection [10]. We found a rapid loss in body mass post challenge for all the strains in hACE2 mice with 2019-nCoV or B.1.617.2 showing 25–30% decrease in body mass and B.1.1.529 showing approximately 10% decrease in body mass when compared to the UI control group (Fig. 2F). The excised lungs from infected hACE2 mice showed prominent features of inflammation and pneumonia as compared to the lungs of UI group (Fig. 2G). However, when compared to the 2019-nCoV-infected group the disease index score of B.1.1529 group was 20–25% lesser than the 2019-nCoV scores (Fig. 2I, J). Lung viral load data from infected groups showed significantly higher viral loads in all the challenged strain groups; however, B.1.1.529 group had approximately two-folds (on log10 scale) lower lung viral load as compared to 2019-nCoV- or B.1.617.2-infected hamsters (Fig. 2H).

## Discussion

Delta variant of the SARS-CoV-2 virus was more pathogenic and virulent than the ancestral virus, leading to reduced real-world vaccine effectiveness [11]. In this study, we demonstrate clinically that the infections attributed to omicron variant were milder than those of delta variant, independent of the immunisation status and previous history of COVID-19. We corroborated these clinical findings with animal experiments to show the lower pathogenicity of omicron variant. The hamsters challenged with omicron SARS-CoV-2 variant (B.1.1.529) demonstrated lower loss of body weight, inflammation and lung viral load compared to the B1.617.2 variant which corroborated with the previously published reports [12, 13].

The COVID-19 surge in the months of March–June 2021 was dominated by the delta variant, with high rates of hospitalization, critical care and deaths [11, 14]. While the reduction in severity with the omicron variant infections as compared to those of delta was consistent with studies from South Africa, the incidence of severe outcomes among omicron infections were lower in our participants as compared with those in South Africa [2, 15, 16]. One of the key reasons to explain the distinct reduction in severe outcomes would be difference in seroprevalence between these populations. The other key reason which could explain the difference in the clinical outcomes was the higher distribution of comorbidities including HIV positivity in the South African population. It is well-documented that patients with co-morbidities tend to have poorer COVID-19 disease prognosis [17–19].

It is also important to consider that the population under study in 2021 had lower vaccination coverage and lower seropositivity. This would have also contributed to the higher proportions of adverse outcomes recorded in 2021 as compared to the omicron-led infections in 2022. Our results after statistical adjustment suggests that the omicron infections could be milder than delta infections independent of vaccination or prior SARS-CoV-2 infection. This is corroborated by the milder pathogenetic changes in the lungs of the animals infected with the omicron variant as compared to the delta variant. This corroboration is a major strength of the study.

We need to be cognisant of a potential limitation. The information on prior infection was collected by history which might have missed some mild or asymptomatic infections. However, we believe that the effect of such a residual confounding on our conclusion would be negligible.

In summary, the omicron infections were inherently milder than the delta variant infections presumably due to the reduced pulmonary pathogenicity of the virus, independent of the protective effect of vaccination and prior SARS-CoV-2 infections. This inherent mildness might have synergistically acted with wider vaccination and thus led to the lower adverse outcomes during the omicron surge globally.

## Data Availability

Data presented in the paper would be made available on request.
